# The population genetics of crypsis in vertebrates: recent insights from mice, hares, and lizards

**DOI:** 10.1038/s41437-019-0257-4

**Published:** 2019-08-09

**Authors:** Rebecca B. Harris, Kristen Irwin, Matthew R. Jones, Stefan Laurent, Rowan D. H. Barrett, Michael W. Nachman, Jeffrey M. Good, Catherine R. Linnen, Jeffrey D. Jensen, Susanne P. Pfeifer

**Affiliations:** 1 School of Life Sciences, Arizona State University, Tempe, AZ USA; 2 School of Life Sciences, École Polytechnique Fédérale de Lausanne, Lausanne, Switzerland; 3 Division of Biological Sciences and Wildlife Biology Program, University of Montana, Missoula, MT USA; 4 Department of Comparative Development and Genetics, Max Planck Institute for Plant Breeding Research, Cologne, Germany; 5 Redpath Museum and Department of Biology, McGill University, Montréal, QC Canada; 6 Department of Integrative Biology and Museum of Vertebrate Zoology, University of California Berkeley, Berkeley, CA USA; 7 Department of Biology, University of Kentucky, Lexington, KY USA

**Keywords:** Rare variants, Evolutionary genetics, Rare variants, Rare variants, Evolutionary genetics

## Abstract

By combining well-established population genetic theory with high-throughput sequencing data from natural populations, major strides have recently been made in understanding how, why, and when vertebrate populations evolve crypsis. Here, we focus on background matching, a particular facet of crypsis that involves the ability of an organism to conceal itself through matching its color to the surrounding environment. While interesting in and of itself, the study of this phenotype has also provided fruitful population genetic insights into the interplay of strong positive selection with other evolutionary processes. Specifically, and predicated upon the findings of previous candidate gene association studies, a primary focus of this recent literature involves the realization that the inference of selection from DNA sequence data first requires a robust model of population demography in order to identify genomic regions which do not conform to neutral expectations. Moreover, these demographic estimates provide crucial information about the origin and timing of the onset of selective pressures associated with, for example, the colonization of a novel environment. Furthermore, such inference has revealed crypsis to be a particularly useful phenotype for investigating the interplay of migration and selection—with examples of gene flow constraining rates of adaptation, or alternatively providing the genetic variants that may ultimately sweep through the population. Here, we evaluate the underlying evidence, review the strengths and weaknesses of the many population genetic methodologies used in these studies, and discuss how these insights have aided our general understanding of the evolutionary process.

## Introduction

The past decade has seen considerable advances in understanding the generation and maintenance of phenotypic diversity. By utilizing the power of high-throughput sequencing, large-scale long-term field studies, and well-established population genetic theory, evolutionary biologists have gained a clearer understanding of the factors underlying adaptive differentiation in natural populations. Many of these insights have stemmed from studies on natural variation in coloration (e.g., Hoekstra 2006; Hubbard et al. 2010).

While animal coloration has been linked to fitness through many avenues (see Cott 1940; Caro 2005; Endler and Mappes 2017; Caro et al. 2017), the evolution of crypsis has received particular interest given its pervasive recurrence across the animal kingdom and its association with the colonization of new environments. Crypsis is the ability of an animal to conceal itself by resembling a sample of the background perceived by visually hunting predators at the time, age, and place where that animal is most frequently preyed upon (Endler 1981). Cryptic background matching is an easily identifiable phenotypic trait that has direct, measurable, and often large fitness consequences (Zimova et al. 2016), making it ideal to study the evolutionary mechanisms underlying adaptation. Indeed, the classical example of adaptive evolution is one of cryptic background matching: industrial melanism in salt and pepper moths in the genus *Biston* (Kettlewell 1955). During the Industrial Revolution in Great Britain, coal soot led to the darkening of previously light colored trees, creating a novel environmental niche for *Biston* moths. Darker moths experienced reduced detection by predators and thus higher survival rates in this new environment (Majerus 1998).

Although numerous studies have focused on the genetic basis of adaptive pigmentation in insects (e.g., Nadeau et al. 2016; Yassin et al. 2016; Linnen et al. 2018), color variation in these systems plays diverse ecological roles and *Biston* remains as the clearest example of cryptic background matching (Cook and Saccheri 2013). By contrast, in vertebrate systems, there are a growing number of studies investigating the genetic basis of crypsis. The best-characterized examples include lizard and mammal populations, usually straddling the edge of a distinct geographical feature formed during the end of the Late Pleistocene. This habitat structure creates a divergent selective environment in which animals have evolved melanin-based coloration matched to their background in order to avoid detection from visually hunting predators. Thus, we now have a collection of studies that involve genetic variation in the same melanin pathway, similar ecological pressures, and adaptation occurring within close physical and temporal proximity. This presents a unique opportunity to compare and contrast evolutionary outcomes in different vertebrate species.

The most compelling studies are those that have integrated ecological, mechanistic, and genomic approaches: working towards establishing the links between phenotype, genotype, and fitness, as well as identifying the signature of selection across the genome (Barrett and Hoekstra 2011). Recent work has benefited from the realization that the inference of selection first requires a robust model of population demography. Factors including fluctuations in population size and/or migration rates may confound the genomic signature of selection, and accounting for these demographic parameters allows not only for the identification of genomic regions which do not conform to neutral expectations, but also for a reduction in false-positive rates (Thornton and Jensen 2007). Following these considerations, a number of studies have successfully connected cryptic phenotypes with an underlying genotype. With demographic models and genetic targets of selection identified, these systems are now shedding light on population genetic questions of long-standing interest, including the prevalence of new vs. standing genetic variants in seeding adaptation, the relative contribution of dominant versus recessive beneficial mutations, the strength of selection experienced by natural populations, and the interplay of gene flow and selection in dictating the pace of adaptive change.

In this review, we summarize examples of cryptic coloration in vertebrates. We restrict the scope to a set of study systems (i.e., rock pocket mice, *Peromyscus* mice, White Sands lizards, and snowshoe hares) in which complementary functional tests combined with detailed population genetic analyses and field-based experimentation have led to a robust understanding of the molecular basis underlying coloration. We discuss the population genetic insights gleaned from such studies, as well as future directions of interest.

## Overview of focal study systems

The study of the focal systems described here has benefited from a long-history of research into the genetic basis of melanin-based pigment. Candidate gene association studies have repeatedly implicated two genes in particular: *Mc1r*, encoding for the melanocortin-1 receptor (MC1R), and *Agouti*, which encodes an MC1R antagonist, the agouti signaling protein (ASP; Hoekstra 2006; Hubbard et al. 2010). Briefly, MC1R acts as a switch to control the type of melanin that is being synthesized in pigment-producing cells (melanocytes). In both mammals and birds, the overall color of an individual is determined by the level of melanin and the ratio of dark (eumelanin) to light (pheomelanin) pigment, which is expressed as dark and light “bands” on hair and feathers. Darker phenotypes result from activation of MC1R which increases eumelanin production, leading to wider eumelanin bands. For lighter phenotypes, ASP acts to antagonize MC1R and either triggers pheomelanin production or shuts down pigment production. Other organisms, including lizards and fish, do not produce pheomelanin and therefore *Mc1r* only affects eumelanin production in these taxa (Hubbard et al. 2010). Below, we begin with a brief overview of each study system.

### Rock pocket mouse (*Chaetodipus intermedius*)

**Fig. 1:**
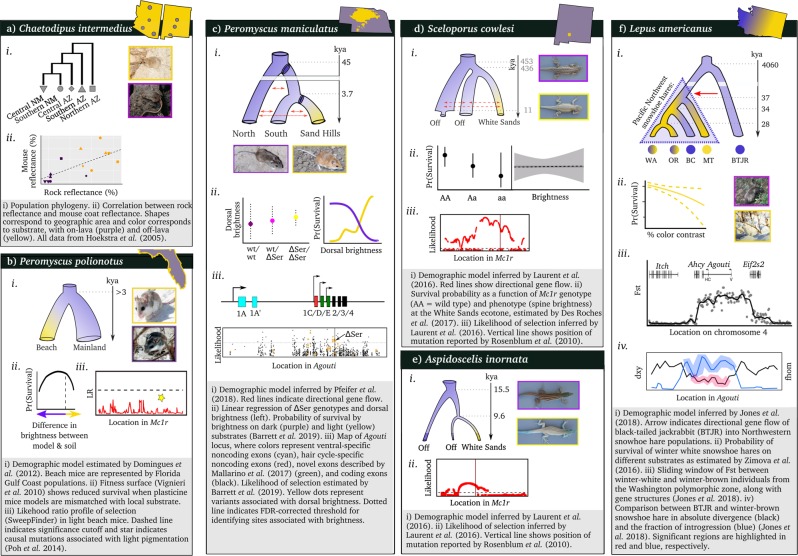
Overview of the focal study systems: **a** *Chaetodipus intermedius*, **b** *Peromyscus polionotus*, **c** *Peromyscus maniculatus*, **d** *Sceloporus cowlesi*, **e** *Aspidoscelis inornata*, and **f** *Lepus americanus*. Each panel depicts the estimated demographic model and the major findings to-date in understanding the genes underlying crypsis. See bottom of each panel for further details. Animal photos from: **a** Nachman et al. 2003, **b** the U.S. Fish and Wildlife Service (*P. polionotus* - light) and the U.S. National Park Service (*P. polionotus* - dark), both released in to the public domain, **c** C. Linnen, **d**, **e** Laurent et al. 2016, **f** Karl Friedrich Herhold (*L. americanus*–light) released under the CC BY 3.0 license, and Walter Siegmund (*L. americanus*–dark) released under the CC BY-SA 3.0 license

The rock pocket mouse (*Chaetodipus intermedius*) represents one of the first vertebrate systems in which the population genetics of cryptic coloration was studied (Fig. 1a). In the deserts of the southwestern United States and adjacent regions in Mexico, this species typically inhabits territories characterized by lightly-colored rocks—a background that is matched by the sandy dorsal pelage of the mice (Benson 1933; Dice and Blossom 1937). Interspersed throughout these lightly-colored regions are patches of black volcanic rock on which populations have evolved darker (melanic) dorsal coats, likely in order to escape detection by visually hunting predators (Nachman et al. 2003). These lava flows are of varying age (some hundreds of thousands of years old, others having formed within the last millennium) and distance (separated by miles of lightly-colored substrate), raising the possibility of convergent evolution of the dorsal color following colonization of these areas (Hoekstra and Nachman 2003). Investigations of the genetic underpinnings of the dorsal pelage color identified genetic variants in *Mc1r* that are in perfect association with the phenotypic changes of the rock pocket mice inhabiting the Pinacate lava flow (Nachman et al. 2003). By contrast, no association was detected between *Mc1r* mutations and the dorsal coat color of mice inhabiting three separate New Mexican lava flows (Hoekstra and Nachman 2003). Consequently, this system has received much attention in understanding the roles of novel parallel mutation versus migration in driving adaptation.

### *Peromyscus* mice: Deer mouse (*P. maniculatus*) and Oldfield mouse (*P. polionotus*)

The present-day diversity and geographic distribution of *Peromyscus*, which is the most abundant mammal in North America, was shaped by events during the Pleistocene. At the end of the last cooling period, glaciers melted, opening up new habitats, increasing sea levels, and depositing sediments in place of glacial cover. These newer substrates often contrast with surrounding soils, in terms of both color and composition, creating divergent selective environments. *Peromyscus* experience a strong selective pressure for cryptic coloration and thus, the colonization of these novel environments was likely facilitated by changes in melanin-based coat coloration (Barrett et al. 2019). Typically inhabiting darkly-colored environments, both the deer mouse (*P. maniculatus*) and the oldfield mouse (*P. polionotus*) have recently colonized lighter soils - the Nebraska Sand Hills, formed by the deposition of wind-blown quartz-sand, Florida’s Gulf coastline, formed from glacial melt, and Florida’s Atlantic coast, formed by shells resulting from rising sea levels (Fig. 1b, c)—causing subsequent shifts towards an overall lighter pelage in these populations (Bedford and Hoekstra 2015). In Florida, multiple independent colonizations of the sandy habitats have been inferred - though the *P. polionotus* populations along the Atlantic and Gulf coasts (often referred to as beach mice) have received the most attention in the scientific literature. Despite the large geographic distance, populations inhabiting light-soil environments are phenotypically more similar to one-another than they are to the nearby dark-soil populations—an observation that raised the possibility of convergent evolution in this system (Steiner et al. 2009). Indeed, different beneficial mutations underlie the same phenotypic adaptation to a light environment in Gulf Coast and Atlantic populations: populations on the Gulf Coast evolved lighter coat coloration through the epistatic interaction between *Agouti* and *Mc1r*, whereas the latter appears to have played no role in the Atlantic coast populations (Fig. 1b; Steiner et al. 2007; Hubbard et al. 2010). Numerous traits (e.g., dorsal color, ventral color, dorsal–ventral boundary, and tail stripe) contribute to the lighter coloration of deer mouse populations residing on the Nebraska Sand Hills, all of which are associated with changes in the *Agouti* gene (Fig. 1c; Linnen et al. 2013; Pfeifer et al. 2018). The genetic underpinnings and the ecological role of crypsis in the Sand Hills populations were established through a combination of robust population genetic inference and direct quantification of the fitness effects of crypsis in the field, making the Nebraska deer mouse the best studied example of cryptic coloration in vertebrates.

### White Sands lizards: Fence lizard (*Sceloporus cowlesi*) and Whiptail lizard (*Aspidoscelis inornata*)

Similar to *Peromyscus*, many North American lizard species were influenced by Pleistocene glaciation. New Mexico’s White Sands—a gypsum dune system formed after the Last Glacial Maximum which contrasts dramatically with the surrounding dark soils of the Chihuahuan Desert—houses multiple populations of lizard species. These include the fence lizard (*Sceloporus cowlesi*) and the whiptail lizard (*Aspidoscelis inornata*), both of which have independently converged upon a cryptic blanched phenotype, generally thought to confer selective advantage through substrate matching to avoid predation (Fig. 1d, e; Rosenblum et al. 2004; Rosenblum et al. 2017). Despite the fact that the same pigmentation gene (*Mc1r*) is associated with a light phenotype, the causal mutation differs in the two species.

### Snowshoe hare (*Lepus americanus*)

The snowshoe hare (*Lepus americanus*) is a boreal North American species that undergoes seasonal coat color change from brown to white to minimize coat color contrast in winter (Fig. 1f). Field experiments have demonstrated that >85% of mortality is related to predation (Hodges 2000) and that rates of survival decreased dramatically in hares mismatched with their background (Zimova et al. 2016), consistent with local selection for seasonal camouflage. Across most of their range, snowshoe hares have white winter coats but in more temperate areas, such as the Pacific Northwest, the probability of molting into a white vs. brown winter coat tracks the gradient from more temperate coastal regions to colder inland environments (Mills et al. 2018). The genetic variation at the pigmentation gene *Agouti* is perfectly associated with winter coat color, with the winter-brown *Agouti* allele appearing sometime after the retreat of the Cordilleran ice sheet. Intriguingly, the *Agouti* allele appears to have originated from an introgressed black-tailed jackrabbit allele, another North American *Lepus* species distributed across prairie-scrub habitats (Jones et al. 2018).

## Population genetic insights

There have been important strides recently in establishing links between genotype, phenotype, and fitness in the above study systems. The population genetic studies discussed here were performed in the wake of numerous candidate gene association and functional studies which uncovered the identity of many of the loci responsible for cryptic coloration. However, as this candidate gene approach is inherently limited to the subset of genes chosen a priori, subsequent genome-wide studies were also employed in order to identify other regions potentially shaped by recent positive selection in an unbiased manner. We describe these findings with an emphasis on the extent to which these crypsis-based results have shed light on topics of general interest in evolutionary genomics.

### The importance of demographic history in identifying loci under selection

**Fig. 2:**
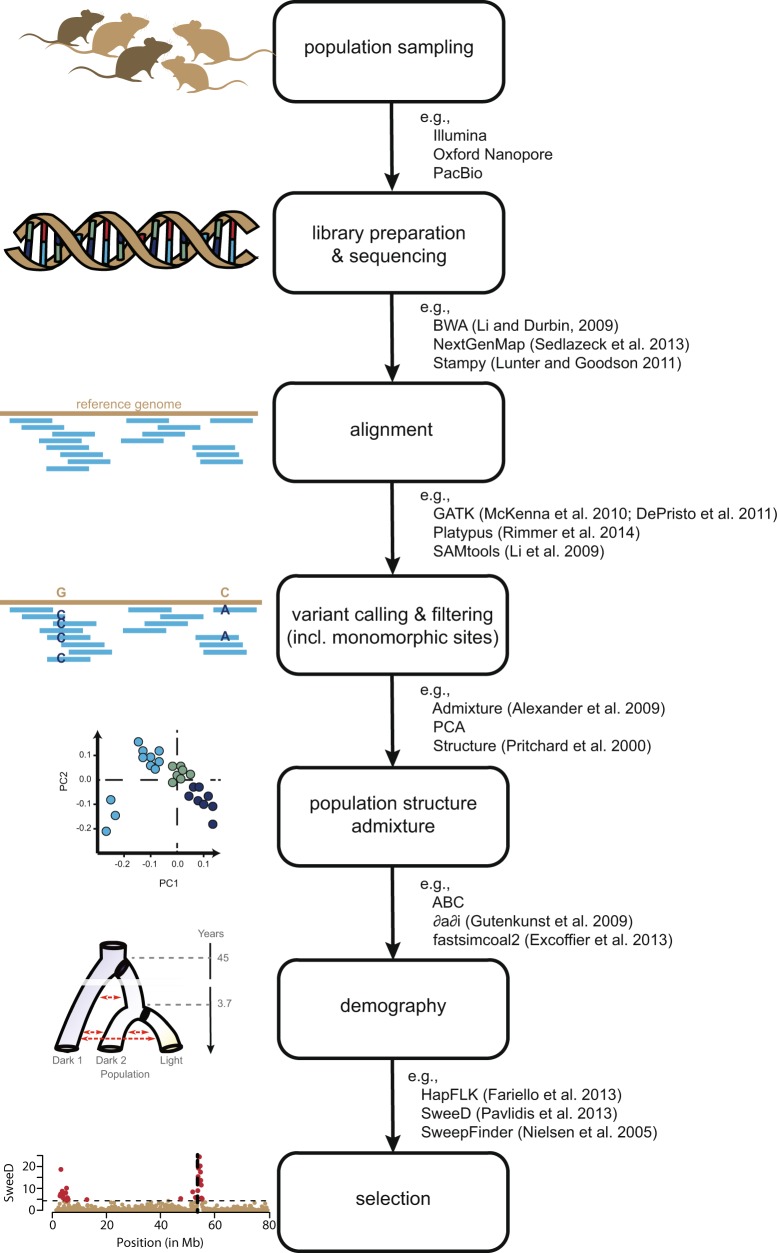
Workflow for identifying beneficial loci. From the top: population sampling is followed by wet-lab procedures, bioinformatic pipelines, and post-assembly analyses. Examples adjacent to black arrows indicate some of the more popular tools of analysis

Selection for crypsis is frequently the result of increased predation following the colonization of a novel habitat—an event which is often associated with demographic shifts including severe population bottlenecks, admixture, and/or changing rates of migration (Lande and Shannon 1996; Lenormand 2002). Thus, following requisite informatic preparations to produce a reliable variant dataset (Meirmans 2015; Pfeifer 2017), it is first necessary to consider the non-equilibrium demographic history of the population in question before attempting to infer the action of selection (Fig. 2). This is crucial as neutral demographic factors may both obscure and mimic the genomic signature of selection (Barton 1998; Thornton and Jensen 2007). For example, both population bottlenecks and selective sweeps may result in reduced genetic variation, a similarly skewed site frequency spectrum (SFS), and distinct patterns of linkage disequilibrium (LD) (Pavlidis and Alachiotis 2017). Numerous methods have been developed to try to distinguish between these signatures (Table 1), though there has been little success in accurately detecting selection in strongly bottlenecked populations (Crisci et al. 2013).

**Table 1:** Brief description of population genetic methods used in the focal studies

Demographic inference				
Method	Data	Definition	Selection	As used in
*δaδi* (Gutenkunst et al. 2009)	Site frequency spectrum (SFS) from one population or the joint frequency spectrum from multiple populations; calculated from neutral polymorphisms	Likelihood of demographic model is calculated using a diffusion approximation	Assumes all SNPs are neutral and unlinked.	Linnen et al. 2013; Laurent et al. 2016; Jones et al. 2018
fastsimcoal2 (Excoffier et al. 2013)	SFS from one population or the joint frequency spectrum from multiple populations; calculated from neutral polymorphisms	Likelihood of demographic model is calculated using coalescent simulations	Assumes all SNPs are neutral and unlinked.	Laurent et al. 2016; Pfeifer et al. 2018

Positive selection				
Method	Data	Definition	Demography	As used in
SweepFinder (Nielsen et al. 2005)	SFS from whole-genome data of one population	For each location in the genome, calculates the likelihood of the observed data under a selective sweep to the likelihood under neutrality. Outputs a composite likelihood ratio (CLR) test statistic. To determine significance, neutral simulations are performed under an inferred demographic model.	Calculates a null SFS from the background SFS, rather than from a strictly equilibrium neutral model	Linnen et al. 2013; Poh et al. 2014; Mallarino et al. 2017
SweeD (Pavlidis et al. 2013)	SFS from whole genome data of one population	Computationally improved version of SweepFinder	Same as above	Laurent et al. 2016; Jones et al. 2018
SweepFinder2 (DeGiorgio et al. 2016)	SFS from whole-genome data of one population	Uses statistical framework of SweepFinder, but accounts for local reductions in diversity caused by purifying selection	Same as above	Pfeifer et al. 2018
ω-statistic (Kim and Nielsen 2004)	SNP data	Calculates the likelihood of the observed data under a selective sweep to the likelihood under neutrality, based on patterns of linkage disequilibrium associated with genetic hitchhiking. To determine significance, neutral simulations are performed under an inferred demographic model.	Demography is not directly accounted for - though demographic simulations should be performed to obtain a neutral distribution of the statistic for comparison to data.	Poh et al. 2014
*Fst* scan	Allele frequency data from at least two populations	Measures genetic differentiation between populations in sliding genomic windows	Implicitly assumes that populations have the same effective population size and are derived independently from the same ancestral population.	Laurent et al. 2016; Jones et al. 2018
HapFLK (Fariello et al. 2013)	Genome-wide SNPs from at least two populations and a population kinship matrix	Calculates a global measure of differentiation for each SNP (FLK) or inferred haplotype (HapFLK) after rescaling frequencies using a population kinship matrix. Genetic differentiation around a selected variant is expected to be larger than expected under drift alone.	Incorporates hierarchical population structure; demographic simulations should be performed to obtain a neutral distribution of the statistic for comparison to data	Pfeifer et al. 2018

An illustrative example of such considerations was highlighted in a recent study contrasting two species of *Peromyscus* mice with differing demographic histories, in which the statistical power to identify mutations underlying the cryptic phenotype was compared (Poh et al. 2014). Prior to this study, the genotypes associated with coat color had been identified, and the targets of selection proposed, in both the Florida beach mice (Vignieri et al. 2010) and the Nebraska Sand Hills mice (Linnen et al. 2013). However, using common statistical methods based on patterns in the SFS and LD (Table 1), the signature of selection was only detected in the Sand Hills mice—a population inferred to have experienced a comparatively mild bottleneck upon colonization compared to the Florida coastal populations (Domingues et al. 2012), resulting in a relatively larger (×200) effective population size. By simulating selective sweeps within the context of these demographic histories, Poh et al. (2014) demonstrated that the more severe population bottleneck combined with the smaller effective population size in the Florida population renders the genomic signature of selection unidentifiable (Fig. 1b). In other words, the swept locus would not appear as a meaningful outlier relative to the tails of the distribution produced under neutrality by the bottleneck alone. This work highlights not only the importance of such neutral demographic inference, but also the value of conducting statistical power analyses under these inferred models.

Apart from skews in the frequency spectrum, levels of population differentiation are also frequently used to identify locally adaptive loci (Table 1). Pfeifer et al. (2018) used an approach which incorporates haplotypic information while controlling for hierarchical population structure (HapFLK; Fariello et al. 2013) to identify *Agouti* variants experiencing positive selection in Sand Hills mice. The HapFLK method has higher power to detect selected loci compared to methods based on simpler measures of differentiation (e.g., the *F*_*ST*_ statistic), even in the presence of complex demographic histories. To ensure the statistical validity of their conclusions, null distributions of HapFLK were simulated under their inferred neutral demographic model characterizing the colonization history of the Sand Hills mice. While significant background (neutral) genetic structure was observed between populations on and off the Sand Hills, the high level of differentiation observed at *Agouti* stood as a significant outlier that was demonstrated to be incompatible with neutrality (Fig. 1c).

### The interplay of natural selection and genetic exchange

Understanding the extent to which gene flow hampers or facilitates local adaptation to new environments remains as a fundamental question in evolutionary genetics. Gene flow between locally adapted populations has the potential to break down population differentiation, and thus disrupt the adaptive process if the strength of selection (*s*) is not sufficiently strong (Haldane 1930). Theoretical work suggests that *s* must be much larger than the rate of migration (*m*) if the beneficial allele is to reach fixation (see review of Tigano and Friesen 2016). Gene flow can also introduce locally deleterious mutations, and theory suggests that the fewer loci needed for the locally adapted phenotype, the lower the resulting migration load. From this prediction, it follows that gene flow will affect the genetic architecture of the underlying adaptive trait by resulting in fewer and larger-effect alleles that may be tightly linked (Yeaman and Whitlock 2011).

Interpreting their results in light of this classical theory (i.e., Haldane 1930), Pfeifer et al. (2018) estimated a migration threshold below which a locally beneficial mutation may be maintained in the Sand Hills mouse population, and then compared this to empirical rates of gene flow inferred under their demographic model. They found that the rate of migration from ancestral dark populations was indeed sufficiently large to prevent the fixation of mutations underlying the light phenotype, while not large enough to eliminate them. In that sense, gene flow is acting to constrain adaptation in the Sand Hills mice. They also examined the genetic architecture of the trait itself: the majority of color variation observed between populations can be explained by *Agouti* variants (e.g., 69% in dorsal brightness) and the majority of genetic variance can be explained by major effect mutations (e.g., 83% for dorsal brightness) localized within a 100 kb region of *Agouti*. Thus, the causal mutations indeed appear to be few in number, of large effect, and in close genomic proximity.

Contrary to the above example, the exchange of genetic material may seed adaptation by providing novel genetic variation at the edge of a species’ range (Pfennig et al. 2016). While gene flow from the center of the range can inundate peripheral populations with maladapted alleles, introgression from different sympatric species can lead to the acquisition of key traits that are already adaptive in the new environment. Building upon previous evidence of interspecific mitochondrial introgression (Cheng et al. 2014; Melo-Ferreira et al. 2014), Jones et al. (2018) reconstructed phylogenetic relationships and conducted tests of genome-wide introgression, with the *Agouti* interval having the strongest signature of introgression between snowshoe hares and black-tailed jackrabbits (Fig. 1f). Furthermore, reduced divergence between these two species at the *Agouti* locus, along with its implication in coat color associations, provided strong support for the causative role of *Agouti*. Coalescent simulations demonstrated that the low-level of divergence seen at *Agouti* is best modelled with interspecific gene flow as opposed to strong selection in the ancestral population. Given that winter-brown and winter-white alleles are locally fixed, despite high rates of gene flow between snowshoe hare populations, seasonal camouflage is likely under strong local selection, consistent with expectations (Zimova et al. 2016). This system therefore provides evidence that hybridization can accelerate adaptation to novel environments by providing novel variants at a greater rate than mutation alone.

In the case of a single species with a large range consisting of patchy habitats, whether adaptation occurs through novel mutations or from gene flow/migration is a function of geographic distance (Ralph and Coop 2010). Populations residing in distant patches are expected to adapt through independent mutations, whereas populations in nearby patches are expected to share alleles. Determining the distance where mutation prevails over gene flow depends, in part, on how maladapted migrants are to the intervening environment: if migrants are well-adapted to the intermediate environments then they will arrive at new patches more quickly than novel mutations can arise. Ralph and Coop (2015) recently developed a mathematical model to derive this expected distance, using the rock pocket mouse as an empirical example. The patches of lava flow that this species inhabit are dispersed throughout the southwest, ranging from between 50–400 km apart. On the Pinacate lava flow in Arizona, which is over a million years old, dark coat color is due to an *Mc1r* allele not found in other lava flow populations in New Mexico, suggesting that the Pinacate adaptation has evolved independently (Nachman et al. 2003). On a younger lava flow in New Mexico (Carrizozo), which is less than 1000 years old, the rock pocket mouse population has also evolved dark coat color. The Carrizozo population is ~100 km from a dark population inhabiting another, older lava flow (Armendaris). Rough calculations suggest that 500–1000 dark alleles may have been introduced to Carrizozo from Armendaris during this time, implicating the potential role of migration in rapid adaptation (Hoekstra et al. 2005). Following this, Ralph and Coop (2015) found that the probability that adaptation occurred through novel parallel mutation increases over a scale of tens to hundreds of kilometers between patches, a range encompassing nearly all lava flows in this region. However, whether the signature of migration is detectable depends on how recombination has broken down the shared haplotype—with the haplotype length being inversely proportional to the number of generations that the beneficial allele spent transiting from one patch to another, which in turn depends upon the dispersal distance relative to the strength of selection against dark coats between patches (see their Fig. 7). Given these findings, Ralph and Coop (2015) demonstrated that for a population like that on the Pinacate lava flow, it would be difficult to differentiate such a model as the length of the shared migrant haplotype would be expected to be short.

Thus, while important questions remain, these systems are proving highly fruitful for evaluating both classic and newly developed theoretical expectations pertaining to the interplay of selection and gene flow in a natural population setting.

### The tempo and mode of adaptation

Given the extensive inference performed, these systems also provide insight in to the respective ages of selective relative to demographic events, as well as the starting frequencies at which beneficial mutations began experiencing selective pressures.

Tempo—the inferred population history can provide important insight regarding the colonization event itself. For example, multiple best-fit demographic models were inferred from empirical Sand Hills mice data (Pfeifer et al. 2018). To distinguish amongst these, inferred demographic parameters were compared against geological information, and data were simulated under the best-fit model so as to ensure that it could recapitulate the observed levels and patterns of genetic variation. Together, these analyses supported a colonization of the Sand Hills from the south approximately 4000 years ago, an estimate that is nearly half the age of the Sand Hills itself. Such a result favors a model of mutation-limited adaptation resulting in hard selective sweeps, as is the case in many of the focal crypsis study systems discussed here.

The estimated age of the beneficial allele can also provide information about the adaptive process, particularly when estimates of population divergence do not conclusively support any single demographic scenario. In the White Sands lizards, population divergence estimates suggest differing times of colonization relative to its geological formation (<6000 years ago; Laurent et al. 2016). Whereas the whiptail experienced a recent split (~4500 years ago), the fence lizard populations diverged earlier (~7400 years ago; Fig. 1d, e). However, the estimated age of the beneficial allele in both whiptail (900 years) and fence lizard (1200 years) post-dates the formation of the White Sands. Together, these results support a more recent colonization by the whiptail, and further highlight the fact that when multiple species have evolved similar cryptic phenotypes in the same environment, they neither necessarily share the same colonization history nor the same underlying genetic variants.

Mode—the origin of beneficial mutations has important implications for understanding constraints on the process of adaptation. The classic model for a selective sweep is one in which selection targets a single newly arising or rare beneficial mutation that then rises to fixation, resulting in a single haplotype at the site of selection (known as a hard selective sweep). Alternatively, selection could target common standing genetic variation that had previously segregated neutrally or was at mutation-selection balance, potentially resulting in multiple haplotype backgrounds at the site of selection (known as a soft selective sweep; Hermisson and Pennings 2005). Whether positive selection more frequently targets rare or common variants has become a point of contention in evolutionary genetics (e.g., Jensen 2014). Importantly, while there are multiple instances of selection acting on standing variation (indeed all selection on recessive alleles would be of this variety by definition), there are few convincing examples from empirical data in which selection acted upon a standing variant of sufficiently high frequency to result in a soft rather than a hard sweep (Harris et al. 2018).

These models have been investigated to varying degrees in the systems discussed here. In the Pinacate lava flow population of rock pocket mice, only one polymorphic site is variable among dark haplotypes, despite there being 17 variable sites among light haplotypes (Nachman et al. 2003). Additionally, the beneficial *Mc1r* allele identified on Pinacate was not sampled on any of the three other lava flow populations or two off-lava flow populations distant from Pinacate (Hoekstra and Nachman 2003). Similarly, the light allele was not sampled in the ancestral populations of the two White Sands lizard populations (Laurent et al. 2016). Additional support for a hard sweep in both lizard species arises from the age of the beneficial allele, estimated to be considerably younger than the timing of the shift in selection pressure. In contrast to the fence lizard, the blanched coloration in the whiptail is caused by a recessive allele (Rosenblum et al. 2010), and thus necessarily the product of selection on standing variation. Yet despite this, a hard sweep pattern is nonetheless supported, consistent with the expectation of selection acting on the segregating variant while at low frequency (Orr and Betancourt 2001). Additionally, owing to its initial rarity in the population, only a single haplotype of the migrant black-tailed jackrabbit allele has reached fixation in snowshoe hare populations (Jones et al. 2018). Finally, the Sands Hills mice represent the most well-supported case where, in addition to all lines of evidence detailed above, the strongly negative fitness effects of the light allele in the ancestral dark-soil habitat have been demonstrated (Barrett et al. 2019)—suggesting that the beneficial derived allele was highly unlikely to have segregated at any appreciable frequency in the ancestral population.

In sum, multiple lines of evidence support a model of positive selection acting on rare rather than common variants in these systems: (1) beneficial, crypsis-related variants often appear on single haplotypes, consistent with a hard sweep model; (2) these variants are generally not found to be segregating in the ancestral environment, as may be expected if they were present as high-frequency neutral alleles prior to the environmental shift; (3) in fragmented habitats, the genotypes underlying the shared phenotype are generally different, suggesting that these populations were not ‘seeded’ with a common, ancestral variant; and (4) the inferred ages of the beneficial mutations are generally younger than the geological age of the altered environment, indicating that the variant was not segregating at the time of the shift (and thus not available for selection to act upon immediately).

### Impact of dominance

It has traditionally been argued that dominant alleles are more likely to contribute to adaptation than recessives due to their relative probabilities of fixation (Haldane 1927). Since dominant alleles may be immediately visible to selection, they are less likely to be lost by genetic drift. These expected differences in the visibility of dominant vs. recessive alleles lead to differing trajectories, with recessive alleles drifting at low frequency followed by a rapid transit time to fixation, and dominant alleles quickly reaching high frequency but then only achieving fixation via genetic drift (see Fig. 2a of Teshima and Przeworski 2006). Given these different trajectories, much of the sojourn time of a recessive mutation is expected to occur when the allele is at low frequency, allowing for a comparatively increased opportunity for the beneficial allele to recombine on to other genetic backgrounds.

The differing dominance patterns observed in White Sands lizards presented Laurent et al. (2016) with a unique opportunity to explore these theoretical predictions. By examining diversity at linked neutral sites from White Sands lizard populations, they confirmed the theoretical findings of Teshima and Przeworski (2006): closer to the selected site, there is a greater reduction in genetic diversity when the beneficial variant is recessive (whiptail lizard), but a wider reduction in diversity when dominant (fence lizard). Given these findings, it is perhaps unsurprising that these two lizards are characterized by different divergence histories: post-divergence the fence lizard population experienced continued gene flow, whereas the whiptail did not (Laurent et al. 2016). Indeed, the bias against the establishment of recessive alleles is exacerbated by gene flow, with theory suggesting that recessive alleles cannot establish when a population inhabiting a new divergent environment is constantly inundated by maladapted alleles from the ancestral range (Orr 2010). Thus, the recessive light allele would be unlikely to have fixed on the White Sands, if it were not for isolation in the absence of gene flow.

Considering the other crypsis systems discussed, Haldane’s view appears to be generally, but not universally, supported. The light *Agouti* allele studied by Linnen et al. (2009) is dominant to the ancestral dark allele in deer mice and the dark *Mc1r* alleles are dominant to the ancestral light allele in rock pocket mice (Nachman et al. 2003). However, in the case of snowshoe hares, the recessive dark allele is derived (with respect to snowshoe hares) with no evidence for strongly reduced gene flow or historic structure between phenotypes (Jones et al. 2018).

### The functional basis of adaptation

Whether adaptive evolution more commonly targets protein-coding or regulatory genes (i.e., those that alter the amount, timing, and location of protein production) is of great interest because of their varying evolutionary implications, including the degree of pleiotropic effects (see review of Stern and Orgogozo 2008). While all known *Mc1r* mutations occur in protein coding regions, there are examples of *Agouti* mutations in both coding and regulatory regions (Hubbard et al. 2010). Following expectations, mutations in *Mc1r* lead to one or more (different) derived amino acid replacements in both the White Sands lizards (Rosenblum et al. 2010) and the rock pocket mice inhabiting the Pinacate lava flow (Nachman et al. 2003), while *cis*-regulatory variation in *Agouti* contributes to cryptic seasonal phenotypes in snowshoe hares (Jones et al. 2018). Both regulatory and structural changes play a role in the light coat color of beach mice on Florida’s Gulf coast and Sand Hills deer mice (Steiner et al. 2007).

While *cis-*regulatory changes have received the most attention, other types of regulatory genes have the potential to alter mRNA processing. Through alternative splicing or alternative transcription initiation or termination sites, a single protein-coding gene can produce multiple isoforms. This has the downstream effect of increasing proteomic diversity and, because of this, such changes in mRNA processing are thought to play an important role in generating phenotypic diversity in vertebrates (Barbosa-Morais et al. 2012). In addition, mounting evidence suggests that splicing diverges more rapidly than gene expression in vertebrates (Merkin et al. 2012), highlighting its potential importance in the rapid evolution of adaptive traits over short timescales. Owing to *Agouti* having served as a model for isoform regulation (Vrieling et al. 1994) and its role in the repeated evolution of cryptic coloration in *Peromyscus*, Mallarino et al. (2017) investigated the role of isoform regulation in deer mice and oldfield mice in adapting to lighter soils. They found that not only have these light populations converged on the same gene, but they have independently converged on upregulation of the same isoform (Fig. 1c)—perhaps driven by selection for the molecular mechanism resulting in the greatest agouti protein production (Mallarino et al. 2017).

## Challenges in connecting genotype to phenotype to fitness

Establishing the adaptive consequences of crypsis requires connecting genotype to phenotype to fitness. An adaptive allele is one which has known functional effects on a phenotypic trait that in turn has a direct effect on fitness (Barrett and Hoekstra 2011). Most of the studies described above either link genotype to fitness through genome-wide scans for selection, or genotype to phenotype through genetic mapping of phenotypic traits. However, connecting all three requires field experiments in which selection on genes with known phenotypic effects can be directly measured.

There are many ways to quantify survival in the field, each methodology having its own requirements and challenges, and these have met with varying degrees of success. A recent study utilizing radiotelemetry directly quantified the fitness costs of background mismatch in natural populations of snowshoe hares, finding that the weekly survival of mismatched hares decreases by 4–7% (Zimova et al. 2016). While radio-collaring permits the study of freely ranging wild animals in their natural habitats, they may be ill-suited to animals with particular life history traits and the collars themselves may confound results by modifying behavior, physiology, and ecology (Barron et al. 2010). Field enclosures are an alternative, however these may not accurately recapitulate natural conditions. In a recent enclosure study on *Holbrookia*—a third lizard species that has evolved blanched coloration on the White Sands—color was not detected as having a significant effect on survival (Hardwick et al. 2015). A number of additional potential issues were raised by the authors of the study, including low statistical power and variations in selection on sex or life stage. Other studies have implemented model-based field experiments to circumvent such issues. These experiments have provided evidence for the selective advantage of crypsis in both oldfield (Vignieri et al. 2010) and deer mice (Linnen et al. 2013). However, as both were conducted using clay models rather than living organisms, they remain a step removed from providing a direct relation to evolutionary change.

While testing cause and effect relationships might demonstrate that a particular trait affects survival, they are not necessarily informative about how genetic variation evolves in response to selective pressures. Instead, if the functional basis of the adaptive trait is known, it is possible to test whether genetic variation evolves in an expected direction by sampling populations at multiple time-points (see review of Habel et al. 2014). In this manner, Barrett et al. (2019) performed a large-scale field experiment to directly estimate the degree to which predation drives changes in allele frequency at *Agouti* in Nebraskan deer mice populations over 14 months. Mice with darker coats were found to have a higher survival probability in dark-soil enclosures and this differential survival resulted in a significant shift in *Agouti* allele frequencies through time. While low statistical power prevented parallel discovery in the light sands enclosures, the phenotype was functionally validated in transgenic mice. Through changes in protein binding properties, a serine deletion in the derived *Agouti* allele leads to decreased production in pheomelanin, causing an increase in dorsal brightness (light coat color). Together, these findings provide direct evidence for divergent natural selection favoring locally adapted pigment phenotypes. Promisingly, with recent advances in DNA extraction and sequencing techniques, it may be increasingly possible to obtain time-sampled allele frequency data through the utilization of natural history collections, even in the absence of such large-scale field experiments (Bi et al. 2013; 2019).

Translating estimates of survival in the field into actual individual and population-level fitness remains a major challenge. Accurate estimates of population growth rates require knowledge of survival and reproduction across different life stages. Snowshoe hares represent one of the few systems in which this type of information has been gathered. After calculating survival costs of snowshoe hare coat color mismatch, Zimova et al. (2016) predicted how waning snow cover due to climate change would affect future mismatch frequency and projected forward. They also translated these differential mortality rates into a predicted population growth rate to demonstrate that selection against mismatch may result in drastic population declines.

Even in the most promising circumstances, the link between genotype, phenotype, and fitness can be difficult to establish. Despite previous evidence that mutations in *Mc1r* are associated with the blanched coloration of fence lizards, a sampling of the White Sands ecotone (transitional habitat with a gradient of dark to light soils) did not recapitulate these results (Des Roches et al. 2017). Furthermore, over the past decade, the frequency of the light *Mc1r* allele has decreased, likely due to a lower survival rate of individuals carrying this allele (Fig. 1d, e). These results may stem from the interplay of ongoing gene flow and selection (Des Roches et al. 2017), as rates of gene flow are likely to be higher in transitional habitats, especially if selection pressures also transition between habitats (e.g., more vegetation provides more cover for individuals mismatched with background). Alternatively, contemporary selective pressures may be different from historical ones: due to anthropogenic factors, the density of avian predators has decreased in recent years. Identifying these differences is particularly important for studies in which contemporary processes are the focal interest (e.g., human-mediated environmental changes).

Another potential explanation from the unexpected fence lizard results may involve phenotypic plasticity (i.e., the ability to produce multiple phenotypes in different environments from a single genotype). By changing the distribution of phenotypes, plastic responses may alter the direction and intensity of selection, and understanding the role of phenotypic plasticity is thus necessary for completing the genotype to phenotype to fitness map. One of the few examples of plasticity preceding adaptation through crypsis in wild populations is the side-blotched lizard (*Uta stansburiana*; Corl et al. 2018). All *Uta* show some degree of phenotypic plasticity in coloration, however, when wild-caught *Uta* were housed on dark soils in the laboratory, the *Uta* collected from the California’s Pisgah lava flow turned significantly darker than those collected from surrounding lighter areas. Two *Mc1r* related genes (i.e., PREP and PRKAR1A, both known regulators of melanin production) show elevated divergence between lizards on and off the lava flow and are associated with increased pigmentation in hatchling lizards. Since plasticity itself is thought to be the target of selection in *Uta*, to establish the genotype to phenotype link future studies will need to assess the joint contribution of these factors.

## Conclusions

Within the context of Fisher’s (1930) Geometric Model, one may expect that the colonization of a novel environment (i.e., the displacement of a population from a fitness optimum) may allow for the possibility of large-effect beneficial mutations early in the adaptive processes. However, such colonization events are also likely to be associated with important demographic changes (such as radical population size changes)—and gene flow may serve to either slow or speed the approach to the optimum. Distinguishing these processes, and quantifying their interplay, remains a central focus of population genetic theory and statistical method development. Importantly, crypsis in vertebrates provides a prime research area to apply these developments, and to directly study these evolutionary processes in natural populations. This is particularly owing to the repeated evolution of similar cryptic phenotypes upon exposure to similar selective pressures, across a wide range of species. Such convergence is also apparent at the level of genes, with numerous studies implicating *Mc1r* and/or *Agouti*, but not at the level of specific mutations. Evidence to date largely supports a model in which the variants underlying crypsis do not pre-date the shift in selective pressure, and thus the timing of phenotypic change is limited by the slow, subsequent input of the necessary genotypic changes.

This research also suggests a number of fruitful future directions. With regards to empirical studies, the picture emerging is one in which *Agouti* and *Mc1r* are highly important, but not necessarily the exclusive players (e.g., Corl et al. 2018). As such, expanding the search radius to whole genomes, rather than focusing on a pre-determined set of candidate loci, will likely provide additional insights into the genetic underpinnings of cryptic coloration. Furthermore, while some recent experimental field studies have successfully connected crypsis with the (long hypothesized) avoidance of predators (e.g., Barrett et al. 2019), others have suggested the likely presence of alternative, potentially even stronger, pressures (e.g., Des Roches et al. 2017). Thus, expanding studies to further quantify the fitness pressures acting on this phenotype in each particular system will likely provide a more complete picture. Finally, with regards to statistical development, while the importance of accounting for the effects of neutral processes has been clearly shown, current methods require the presence of neutral, unlinked regions (e.g., intergenic regions distant from any functional elements) in order to first estimate a demographic model. That model is then fixed for the purposes of scanning for selective effects around candidate loci. While this approach may indeed significantly reduce false-positive rates, the identification of strictly neutral regions is both challenging and difficult to verify, particularly in non-model organisms where high-quality reference assemblies and gene annotations are often lacking. In order to avoid this two-step process requiring separate types of genomic data (i.e., putatively neutral and putatively selected), calls have recently been made to develop methods capable of jointly inferring demographic and selective effects (Comeron 2017; Jensen et al. 2019). Importantly, such approaches could allow researchers to naively scan newly-emerging systems for localized selective sweep patterns, even in the absence of a well-annotated reference genome.

## References

[CR1] Alexander DH, Novembre J, Lange K (2009). Fast model-based estimation of ancestry in unrelated individuals. Genome Res.

[CR2] Barbosa-Morais NL, Irimia M, Pan Q, Xiong HY, Gueroussov S, Lee LJ (2012). The evolutionary landscape of alternative splicing in vertebrate species. Science.

[CR3] Barrett RDH, Hoekstra HE (2011). Molecular spandrels: tests of adaptation at the genetic level. Nat Rev Genet.

[CR4] Barrett RDH, Laurent S, Mallarino R, Pfeifer SP, Xu CCY, Foll MF (2019). Linking a mutation to survival in wild mice. Science.

[CR5] Barron DG, Brawn JD, Weatherhead PJ (2010). Meta-analysis of transmitter effects on avian behaviour and ecology. Methods Ecol Evol.

[CR6] Barton NH (1998). The effect of hitch-hiking on neutral genealogies. Genet Res.

[CR7] Bedford NL, Hoekstra HE (2015). *Peromyscus* mice as a model for studying natural variation. eLife.

[CR8] Benson SB (1933). Concealing coloration among some desert rodents of the Southwestern United States. Univ Calif Publ Zool.

[CR9] Bi K, Linderoth T, Vanderpool D, Good JM, Nielsen R, Moritz C (2013). Unlocking the vault: next-generation museum population genomics. Mol Ecol.

[CR10] Bi K, Linderoth T, Singhal S, Vanderpool D, Patton JL, Nielsen R, Moritz C, Good JM (2019). Temporal genomic contrasts reveal rapid evolutionary responses in an alpine mammal during recent climate change. PLoS Genet.

[CR11] Caro T (2005). The adaptive significance of coloration in mammals. Bioscience.

[CR12] Caro T, Stoddard MC, Stuart-Fox D (2017). Animal coloration research: why it matters. Philos Trans R Soc B.

[CR13] Cheng E, Hodges KE, Melo-Ferreira J, Alves PC, Mills LS (2014). Conservation implications of the evolutionary history and genetic diversity hotspots of the snowshoe hare. Mol Ecol.

[CR14] Comeron JM (2017). Background selection as a null hypothesis in population genomics: insights and challenges from Drosophila studies. Philos Trans R Soc B.

[CR15] Cook LM, Saccheri IJ (2013). The peppered moth and industrial melanism: evolution of a natural selection case study. Heredity.

[CR16] Corl A, Bi K, Luke C, Challa AS, Stern AJ, Sinervo B, Nielsen R (2018). The genetic basis of adaptation following plastic changes in coloration in a novel environment. Curr Biol.

[CR17] Cott HB (1940) Adaptive coloration in animals. Oxford University Press, Oxford

[CR18] Crisci JL, Poh YP, Mahajan S, Jensen JD (2013). The impact of equilibrium assumptions on tests of selection. Front Genet.

[CR19] DeGiorgio M, Huber CD, Hubisz MJ, Hellmann I, Nielsen R (2016). SweepFinder2: increased sensitivity, robustness, and flexibility. Bioinformatics.

[CR20] DePristo M, Banks E, Poplin R, Garimella KV, Maguire JR, Hartl C (2011). A framework for variation discovery and genotyping using next-generation DNA sequencing data. Nat Genet.

[CR21] Des Roches S, Sollmann R, Calhoun K, Rothstein AP, Rosenblum EB (2017). Survival by genotype: patterns at *Mc1r* are not black and white at the White Sands ecotone. Mol Ecol.

[CR22] Dice LR, Blossom PM (1937). Studies of mammalian ecology in south-western North America with special reference to the colors of desert animals. Carne Inst Wash Publ.

[CR23] Domingues VS, Poh YP, Peterson BK, Pennings PS, Jensen JD, Hoekstra HE (2012). Evidence of adaptation from ancestral variation in young populations of beach mice. Evolution.

[CR24] Endler JA (1981). An overview of the relationships between mimicry and crypsis. Biol J Linn Soc.

[CR25] Endler JA, Mappes J (2017). The current and future state of animal coloration research. Philos Trans R Soc B.

[CR26] Excoffier L, Dupanloup I, Huerta-Sanchez E, Sousa VC, Foll M (2013). Robust demographic inference from genomic and SNP data. PLoS Genet.

[CR27] Fariello MI, Boitard S, Naya H, San Cristobal M, Servin B (2013). Detecting signatures of selection through haplotype differentiation among hierarchically structured populations. Genetics.

[CR28] Fisher RA (1930) The genetical theory of natural selection: a complete variorum edition. Oxford University Press, Oxford

[CR29] Gutenkunst RN, Hernandez RD, Williamson SH, Bustamante CD (2009). Inferring the joint demographic history of multiple populations from multidimensional SNP frequency data. PLoS Genet.

[CR30] Habel JC, Husemann M, Finger A, Danley PD, Zachos FE (2014). The relevance of time series in molecular ecology and conservation biology. Biol Rev Camb Philos Soc.

[CR31] Haldane JBS (1927). A mathematical theory of natural and artificial selection. Math Proc Camb Philos Soc.

[CR32] Haldane JBS (1930). A mathematical theory of natural and artificial selection. VI. Isolation. Proc Camb Philos Soc.

[CR33] Hardwick KM, Harmon LJ, Hardwick SD, Rosenblum EB (2015). When field experiments yield unexpected results: lessons learned from measuring selection in White Sands lizards. PLoS ONE.

[CR34] Harris RB, Sackman AM, Jensen JD (2018). On the unfounded enthusiasm for soft selective sweeps II: examining recent evidence from humans, flies, and viruses. PLoS Genet.

[CR35] Hermisson J, Pennings PS (2005). Soft sweeps: molecular population genetics of adaptation from standing genetic variation. Genetics.

[CR36] Hodges KE (2000) Ecology of snowshoe hares in southern boreal and montane forests. In: Ruggiero L, Aubry KB, Buskirk SW, Koehler GM, Krebs CJ, McKelvey KS, Squires JR (eds) Ecology and Conservation of Lynx in the United States. University Press of Colorado, Boulder p 163–206

[CR37] Hoekstra HE (2006). Genetics, development and evolution of adaptive pigmentation in vertebrates. Heredity.

[CR38] Hoekstra HE, Krenz JG, Nachman MW (2005). Local adaptation in the rock pocket mouse (*Chaetodipus intermedius*): natural selection and phylogenetic history of populations. Heredity.

[CR39] Hoekstra HE, Nachman MW (2003). Different genes underlie adaptive melanism in different populations of rock pocket mice. Mol Ecol.

[CR40] Hubbard JK, Uy JAC, Hauber ME, Hoekstra HE, Safran RJ (2010). Vertebrate pigmentation: from underlying genes to adaptive function. Trends Genet.

[CR41] Jensen JD (2014). On the unfounded enthusiasm for soft selective sweeps. Nat Commun.

[CR42] Jensen JD, Payseur BA, Stephan W, Aquadro CF, Lynch M, Charlesworth D, Charlesworth B (2019). The importance of the Neutral Theory in 1968 and 50 years on: a response to Kern & Hahn 2018. Evolution.

[CR43] Jones MR, Mills LS, Alves PC, Callahan CM, Alves JM, Lafferty DJR (2018). Adaptive introgression underlies polymorphic seasonal camouflage in snowshoe hares. Science.

[CR44] Kettlewell HBD (1955). Selection experiments on industrial melanism in the Lepidoptera. Heredity.

[CR45] Kim Y, Nielsen R (2004). Linkage disequilibrium as a signature of selective sweeps. Genetics.

[CR46] Lande R, Shannon S (1996). The role of genetic variation in adaptation and population persistence in a changing environment. Evolution.

[CR47] Laurent S, Pfeifer SP, Settles ML, Hunter SS, Hardwick KM, Ormond L (2016). The population genomics of rapid adaptation: disentangling signatures of selection and demography in white sands lizards. Mol Ecol.

[CR48] Lenormand T (2002). Gene flow and the limits to natural selection. Trends Ecol Evol.

[CR49] Li H, Durbin R (2009). Fast and accurate short read alignment with Burrows-Wheeler transform. Bioinformatics.

[CR91] Li H, Handsaker B, Wysoker A, Fennell T, Ruan J, Homer N (2009). The Sequence Alignment/Map format and SAMtools. Bioinformatics.

[CR50] Linnen CR, Kingsley EP, Jensen JD, Hoekstra HE (2009). On the origin and spread of an adaptive allele in deer mice. Science.

[CR51] Linnen CR, O’Quin CT, Shackleford T, Lindstedt C (2018). Genetic basis of body color and spotting pattern in redheaded pine sawfly larvae (*Neodiprion lecontei*). Genetics.

[CR52] Linnen CR, Poh YP, Peterson BK, Barrett RDH, Larson JG, Jensen JD, Hoekstra HE (2013). Adaptive evolution of multiple traits through multiple mutations at a single gene. Science.

[CR53] Lunter G, Goodson M (2011). Stampy: a statistical algorithm for sensitive and fast mapping of Illumina sequence reads. Genome Res.

[CR54] Majerus MEN (1998) Melanism: evolution in action. Oxford University Press, Oxford

[CR55] Mallarino R, Linden TA, Linnen CR, Hoekstra HE (2017). The role of isoforms in the evolution of cryptic coloration in *Peromyscus* mice. Mol Ecol.

[CR56] McKenna A, Hanna M, Banks E, Sivachenko A, Cibulskis K, Kernytsky A (2010). The Genome Analysis Toolkit: a MapReduce framework for analyzing next-generation DNA sequencing data. Genome Res.

[CR57] Meirmans PG (2015). Seven common mistakes in population genetics and how to avoid them. Mol Ecol.

[CR58] Melo-Ferreira J, Seixas FA, Cheng E, Mills LS, Alves PC (2014). The hidden history of the snowshoe hare, *Lepus americanus*: extensive mitochondrial DNA introgression inferred from multilocus genetic variation. Mol Ecol.

[CR59] Merkin J, Russell C, Chen P, Burge CB (2012). Evolutionary dynamics of gene and isoform regulation in Mammalian tissues. Science.

[CR60] Mills LS, Bragina VE, Kumar AV, Zimova M, Lafferty DJR, Feltner J (2018). Winter coat color polymorphisms identify global hotspots for evolutionary rescue from climate change. Science.

[CR61] Nachman MW, Hoekstra HE, D’Agostino SL (2003). The genetic basis of adaptive melanism in pocket mice. Proc Natl Acad Sci USA.

[CR62] Nadeau NJ, Pardo-Diaz C, Whibley A, Supple MA, Saenko SV, Wallbank RWR (2016). The gene cortex controls mimicry and crypsis in butterflies and moths. Nature.

[CR63] Nielsen R, Williamson S, Kim Y, Hubisz MJ, Clark AG, Bustamante C (2005). Genomic scans for selective sweeps using SNP data. Genome Res.

[CR64] Orr HA (2010). The population genetics of beneficial mutations. Philos Trans R Soc Lond B Biol Sci.

[CR65] Orr HA, Betancourt AJ (2001). Haldane’s sieve and adaptation from the standing genetic variation. Genetics.

[CR66] Pavlidis P, Alachiotis N (2017). A survey of methods and tools to detect recent and strong positive selection. J Biol Res-Thessalon.

[CR67] Pavlidis P, Zivkovic D, Stamatakis A, Alachiotis N (2013). SweeD: likelihood-based detection of selective sweeps in thousands of genomes. Mol Biol Evol.

[CR68] Pfeifer SP (2017). From next-generation resequencing reads to a high-quality variant data set. Heredity.

[CR69] Pfeifer SP, Laurent S, Sousa VC, Linnen CR, Foll M, Excoffier L (2018). The evolutionary history of Nebraska deer mice: local adaptation in the face of strong gene flow. Mol Biol Evol.

[CR70] Pfennig K, Kelly AL, Piece AA (2016). Hybridization as a facilitator of species range expansion. Proc Camb Philos Soc.

[CR71] Poh YP, Domingues VS, Hoekstra HE, Jensen JD (2014). On the prospect of identifying adaptive loci in recently bottlenecked populations. PLoS ONE.

[CR72] Pritchard JK, Stephens M, Donnelly P (2000). Inference of population structure using multilocus genotype data. Genetics.

[CR73] Ralph P, Coop G (2010). Parallel adaptation: one or many waves of advance of an advantageous allele?. Genetics.

[CR74] Ralph PL, Coop G (2015). Convergent evolution during local adaptation to patchy landscapes. PLoS Genet.

[CR75] Rimmer A, Phan H, Mathieson I, Iqbal Z, Twigg SR, Wilkie AO (2014). Integrating mapping-, assembly- and haplotype-based approaches for calling variants in clinical sequencing applications. Nat Genet.

[CR76] Rosenblum EB, Hoekstra HE, Nachman MW (2004). Adaptive reptile color variation and the evolution of the *Mc1r* gene. Evolution.

[CR77] Rosenblum EB, Römpler H, Schöneberg T, Hoekstra HE (2010). Molecular and functional basis of phenotypic convergence in white lizards at White Sands. Proc Natl Acad Sci USA.

[CR78] Rosenblum EB, Parent CE, Diepeveen ET, Noss C, Bi K (2017). Convergent phenotypic evolution despite contrasting demographic histories in the fauna of White Sands. Am Nat.

[CR79] Sedlazeck FJ, Rescheneder F, von Haeseler A (2013). NextGenMap: fast and accurate read mapping in highly polymorphic genomes. Bioinformatics.

[CR80] Steiner CC, Römpler H, Boettger LM, Schöneberg T, Hoekstra HE (2009). The genetic basis of phenotypic convergence in beach mice: similar pigment patterns but different genes. Mol Biol Evol.

[CR81] Steiner CC, Weber JN, Hoekstra HE (2007). Adaptive variation in beach mice produced by two interacting pigmentation genes. PLoS Biol.

[CR82] Stern DL, Orgogozo V (2008). The loci of evolution: how predictable is genetic evolution?. Evolution.

[CR83] Teshima KM, Przeworski M (2006). Directional positive selection on an allele of arbitrary dominance. Genetics.

[CR84] Tigano A, Friesen VL (2016). Genomics of local adaptation with gene flow. Mol Ecol.

[CR85] Thornton KR, Jensen JD (2007). Controlling the false-positive rate in multilocus genome scans for selection. Genetics.

[CR86] Vignieri SN, Larson JG, Hoekstra HE (2010). The selective advantage of crypsis in mice. Evolution.

[CR87] Vrieling H, Duhl DM, Millar SE, Miller KA, Barsh GS (1994). Differences in dorsal and ventral pigmentation result from regional expression of the mouse agouti gene. Proc Natl Acad Sci USA.

[CR88] Yassin A, Bastide H, Chung H, Veuille M, David JR, Pool JE (2016). Ancient balancing selection at *tan* underlies female colour dimorphism in *Drosophila erecta*. Nat Commun.

[CR89] Yeaman S, Whitlock MC (2011). The genetic architecture of adaptation under migration-selection balance. Evolution.

[CR90] Zimova M, Mills LS, Nowak JJ (2016). High fitness costs of climate change-induced camouflage mismatch. Ecol Lett.

